# Supplementary material for: A Non-stop identity complex (NIC) supervises enterocyte identity and protects from premature aging
Source: eLife. 2021 Feb 25;10:e62312. doi: 10.7554/eLife.62312 (PMC7936876; doi:10.7554/eLife.62312)
Supplement: Transparent reporting form [file elife-62312-transrepform.pdf]

Westbrook Centre
Milton Road
Cambridge CB4 1YG, UK

P 01223 855340
W elifesciences.org
T @elife

eLife's transparent reporting form

We encourage authors to provide detailed information *within their submission* to facilitate the interpretation and replication of experiments. Authors can upload supporting documentation to indicate the use of appropriate reporting guidelines for health-related research (see EQUATOR Network), life science research (see the BioSharing Information Resource), or the ARRIVE guidelines for reporting work involving animal research. Where applicable, authors should refer to any relevant reporting standards documents in this form.

If you have any questions, please consult our Journal Policies and/or contact us: editorial@elifesciences.org.

Sample-size estimation

- You should state whether an appropriate sample size was computed when the study was being designed
- You should state the statistical method of sample size computation and any required assumptions
- If no explicit power analysis was used, you should describe how you decided what sample (replicate) size (number) to use

Please outline where this information can be found within the submission (e.g., sections or figure legends), or explain why this information doesn't apply to your submission:

All the information regarding the design and conductance of experimentation, as well as data analysis is detailed in the manuscript in the text, figure legend and methods. This include, sample size, number of biological repeats, exact transgenic lines used in each experiment, as well as detail information regarding computational bioinformatics statistical methodology. We also listed detail information regarding the specific antibodies used including concentrations. All in all, we hope that this information will assist the community to build upon our findings.

Replicates

- You should report how often each experiment was performed
- You should include a definition of biological versus technical replication
- The data obtained should be provided and sufficient information should be provided to indicate the number of independent biological and/or technical replicates
- If you encountered any outliers, you should describe how these were handled
- Criteria for exclusion/inclusion of data should be clearly stated
- High-throughput sequence data should be uploaded before submission, with a private link for reviewers provided (these are available from both GEO and Array-Express)

Please outline where this information can be found within the submission (e.g., sections or figure legends), or explain why this information doesn't apply to your submission:

eLife Sciences Publications, Ltd is a limited liability non-profit non-stock corporation incorporated in the State of Delaware, USA, with company number 5030732, and is registered in the UK with company number FC030576 and branch number BR015634 at the address Westbrook Centre, Milton Road, Cambridge CB4 1YG.

All the above information regarding replicates can be found in the text under methods, figure legend and results. Detailed experimental design and bioinformatics methodology are under methods. Lists of genes with statistically significant changes are all presented under supplemental tables .

Detailed information including all raw data was uploaded to GEO: Gene expression analysis was deposited at GEO: RNAseq and ATAC-seq data are available at NCBI through the Accession number PRJNA657899 ID: 657899

Link to the the proteomic data set is: ftp://massive.ucsd.edu/MSV000082625. The data is also accessible from: ProteomeXChange accession: PXD010462 http://proteomecentral.proteomexchange.org/cgi/GetDataset?ID=PXD010462. MassIVE | Accession ID: MSV000082625 - ProteomeXchange | Accession ID: PXD010462.

Statistical reporting

- Statistical analysis methods should be described and justified
- Raw data should be presented in figures whenever informative to do so (typically when N per group is less than 10)
- For each experiment, you should identify the statistical tests used, exact values of N, definitions of center, methods of multiple test correction, and dispersion and precision measures (e.g., mean, median, SD, SEM, confidence intervals; and, for the major substantive results, a measure of effect size (e.g., Pearson's r, Cohen's d)
- Report exact p-values wherever possible alongside the summary statistics and 95% confidence intervals. These should be reported for all key questions and not only when the p-value is less than 0.05.

Please outline where this information can be found within the submission (e.g., sections or figure legends), or explain why this information doesn’t apply to your submission:

(For large datasets, or papers with a very large number of statistical tests, you may upload a single table file with tests, Ns, etc., with reference to sections in the manuscript.)

All statistical information is within the figure legend and “method” section.

Group allocation

- Indicate how samples were allocated into experimental groups (in the case of clinical studies, please specify allocation to treatment method); if randomization was used, please also state if restricted randomization was applied
- Indicate if masking was used during group allocation, data collection and/or data analysis

Please outline where this information can be found within the submission (e.g., sections or figure legends), or explain why this information doesn’t apply to your submission:

eLife Sciences Publications, Ltd is a limited liability non-profit non-stock corporation incorporated in the State of Delaware, USA, with company number 5030732, and is registered in the UK with company number FC030576 and branch number BR015634 at the address Westbrook Centre, Milton Road, Cambridge CB4 1YG.

Sample size are indicated in the method section, the specific genotypes are indicated in the results and methods sections and figures and legend to figure.

Additional data files ("source data")

- We encourage you to upload relevant additional data files, such as numerical data that are represented as a graph in a figure, or as a summary table
- Where provided, these should be in the most useful format, and they can be uploaded as "Source data" files linked to a main figure or table
- Include model definition files including the full list of parameters used
- Include code used for data analysis (e.g., R, MatLab)
- Avoid stating that data files are "available upon request"

Please indicate the figures or tables for which source data files have been provided:

Source data are avialble : Figures with sources data 1, 2, 3, 4, 5, 6, 7, 9
Extended data included: Figure 1 source data 2: results of RNAi screen
Figure 3 source data 1: proteomic analysis
Figure 5 source data 1: Non-stop RNA-seq
Figure 5 source data 2: Non-stop ATAC-seq
Figure 5 source data 3: ATAC-seq clusters

Prof. Amir Orian

eLife Sciences Publications, Ltd is a limited liability non-profit non-stock corporation incorporated in the State of Delaware, USA, with company number 5030732, and is registered in the UK with company number FC030576 and branch number BR015634 at the address Westbrook Centre, Milton Road, Cambridge CB4 1YG.
